# Reversal of TMS-induced motor twitch by training is associated with a reduction in excitability of the antagonist muscle

**DOI:** 10.1186/1743-0003-8-46

**Published:** 2011-08-24

**Authors:** Viola Giacobbe, Bruce T Volpe, Gary W Thickbroom, Felipe Fregni, Alvaro Pascual-Leone, Hermano I Krebs, Dylan J Edwards

**Affiliations:** 1 Burke-Cornell Medical Research Institute, White Plains, NY, USA; 2 Center for Neuromuscular and Neurological Disorders, University of Western Australia, Perth, Australia; 3 Berenson-Allen Center for Noninvasive Brain Stimulation, Beth Israel Deaconess Medical Center, Harvard Medical School, Boston, MA, USA; 4 MIT, Boston, MA, USA; 5 Institut Guttmann, Universitat Autonoma de Barcelona, Barcelona, Spain; 6 Laboratory of Neuromodulation, Spaulding Rehabilitation Hospital, Harvard Medical School, Boston, MA, USA

## Abstract

**Background:**

A single session of isolated repetitive movements of the thumb can alter the response to transcranial magnetic stimulation (TMS), such that the related muscle twitch measured post-training occurs in the trained direction. This response is attributed to transient excitability changes in primary motor cortex (M1) that form the early part of learning. We investigated; (1) whether this phenomenon might occur for movements at the wrist, and (2) how specific TMS activation patterns of opposing muscles underlie the practice-induced change in direction.

**Methods:**

We used single-pulse suprathreshold TMS over the M1 forearm area, to evoke wrist movements in 20 healthy subjects. We measured the preferential direction of the TMS-induced twitch in both the sagittal and coronal plane using an optical goniometer fixed to the dorsum of the wrist, and recorded electromyographic (EMG) activity from the flexor carpi radialis (FCR) and extensor carpi radialis (ECR) muscles. Subjects performed gentle voluntary movements, in the direction opposite to the initial twitch for 5 minutes at 0.2 Hz. We collected motor evoked potentials (MEPs) elicited by TMS at baseline and for 10 minutes after training.

**Results:**

Repetitive motor training was sufficient for TMS to evoke movements in the practiced direction opposite to the original twitch. For most subjects the effect of the newly-acquired direction was retained for at least 10 minutes before reverting to the original. Importantly, the direction change of the movement was associated with a significant decrease in MEP amplitude of the antagonist to the trained muscle, rather than an increase in MEP amplitude of the trained muscle.

**Conclusions:**

These results demonstrate for the first time that a TMS-twitch direction change following a simple practice paradigm may result from reduced corticospinal drive to muscles antagonizing the trained direction. Such findings may have implications for training paradigms in neurorehabilitation.

## Background

Human motor control of individual joints involves organized coupling of agonist and antagonist muscles to achieve a desired movement efficiently. During contraction of agonist muscles, the antagonists do not behave passively, but are actively inhibited by central nervous mechanisms [1]. Reciprocal control of antagonistic muscles is critical for execution of coordinated limb movements, and through a mechanism of reciprocal inhibition, the central nervous system ensures that antagonist muscle activity is suppressed during contraction of an agonist [2].

During motor learning, patterns of motor activation are encoded in the brain through distributed networks including motor cortex, deep brain nuclei and the cerebellum [3]. In primary motor cortex (M1) these changes can be probed with mapping techniques showing excitability changes and representational reorganization associated with extensive motor training [4-6], depending on the nature of movements performed during training [7]. These studies have clinical implications since motor training is known to positively influence motor control in neurological patients [8-11], and novel interventions are emerging that actively alter cortical excitability and might interact with training effects [12]. However, the corticomotor excitability changes associated with well-defined, simple training paradigms in healthy humans are poorly understood, particularly those relating to agonist-antagonist muscle pairs.

A single suprathreshold pulse of Transcranial Magnetic Stimulation (TMS) over the hand area of M1 results in a balance of inhibitory and excitatory processes that leads to an observed twitch of the thumb in a consistent direction with each stimulus [13]. Further, a short period of practice with movements in the opposite direction can change the direction of the TMS-induced twitch to that of the practice direction. It remains to be investigated how the relationship between agonist and antagonist muscle activation might lead to this direction change, or if this phenomenon is peculiar to muscles of the thumb.

In the present study we examined in healthy adults whether the direction of TMS-induced wrist movements can be modulated or changed by a short period of simple repetitive wrist training. We proposed to test muscles controlling the wrist that are located in the proximal forearm area and that have a more defined functional agonist-antagonist role. We hypothesized that a short period of repetitive gentle wrist movements in a direction opposite to the initial TMS-twitch direction, with only concentric contraction of the agonist (passive return), would result in a change of twitch direction elicited by TMS, and a corresponding reduction in descending drive to the antagonist muscle.

## Methods

### Subjects

Twenty right-handed healthy volunteers (mean age 28 yrs, range 22-37 yrs) with no history of neurological or psychiatric illness, and no contraindications to TMS, were recruited for the experiment. The subjects were seated comfortably in a chair with their right arm freely hanging to the side in a relaxed posture. All subjects were screened for TMS exclusion criteria and gave their written informed consent before participating. The study was approved by the Institutional Review Board of Burke Rehabilitation Hospital.

### Stimulation set-up

Biphasic single-pulse TMS was delivered through a figure-of-eight-shaped coil (inner diameter: 35 mm, outer diameter: 75 mm, MagVenture), using a MagPro x100 stimulator (Mindcare Co.). To identify the area of stimulation, a tight lycra cap was positioned over the head and the vertex was marked by measuring the mid-point intersection between the nasion-inion and inter-aural lines. Potential stimulus sites were marked on the cap using the vertex as a reference point, in 1-cm steps in the coronal and sagittal planes, over the region of the primary motor cortex. Using a supra-threshold stimulus intensity, the coil was systematically moved over motor cortex to determine the optimal location for eliciting isolated wrist movement, and maximal amplitude motor evoked potentials (MEPs) in both the flexor carpi radialis (FCR) and extensor carpi radialis (ECR) muscles. MEPs were obtained from the FCR and ECR muscles simultaneously. Once the optimal position of the coil was established, it was marked on the cap, to ensure a constant coil placement throughout the experiment.

During stimulation, the center of the coil was placed tangentially to the scalp with the handle pointing posterior and laterally rotated at a 45° angle from the midline, in order to induce a posterior-anterior current flow in the cortical tissue approximately perpendicular to the line of the central sulcus. Focal TMS was delivered to the brain with the target muscles at rest, that is, in the absence of any electromyographic (EMG) activity exceeding a background noise level of 20 μV.

### Recording of EMG and twitch direction

Surface EMG activity was recorded from pre-amplified electrodes (SX230, fixed electrode distance: 20 mm, Biometrics Ltd.) positioned over the muscle belly of the right FCR and ECR muscles. EMG signals were amplified (x1000) at the site and band-pass filtered between 20 and 400 Hz. The signals were collected and digitized at a frequency of 1000 Hz using a Cambridge Electronic Design (CED) 1401 A/D converter and a data-collection program (CED Spike 2), then stored into the computer for further off-line analysis. EMG activity of the training muscles was continuously monitored during practice to provide visual feedback during the experiment and ensure regular contractions during training. In this study the antagonist muscle was defined as the muscle opposing the direction of training.

Resting motor threshold (RMT), defined as the minimum TMS intensity that evoked a MEP of at least 50 μV peak-to-peak amplitude in 6 of 10 trials, was measured for the FCR and ECR in stimulus steps of 1% of maximum stimulator output (MSO). RMT was determined with the wrist resting on the subject's lap, starting at a low intensity and using four stimuli for each 1% increment of stimulator output intensity.

**Figure 1:**
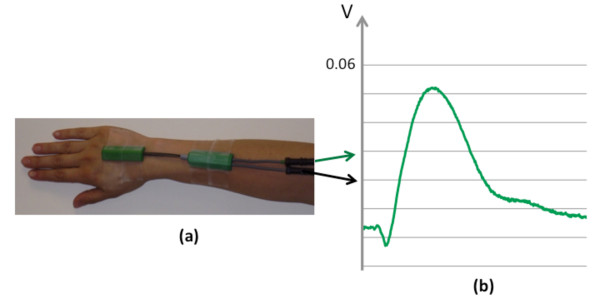
**(a) Two degree-of-freedom optical goniometer fixed to the dorsum of the wrist to measure deflection produced by TMS-induced twitch in the sagittal and coronal plane; (b) An example of goniometer trace as seen in the signal output for sagittal plane**.

A two degree-of-freedom optical goniometer (SG65, max stretch length: 65 mm, Biometrics Ltd.) was positioned on the dorsum of the wrist, aligned in the sagittal plane (Figure 1a), to quantify joint rotations in both the sagittal (wrist flexion or extension) and coronal (wrist ulnar or radial deviation) planes. The output of the goniometer (Figure 1b), together with the EMG readings, was acquired using CED Spike 2 software.

### Experimental design

**Figure 2:**
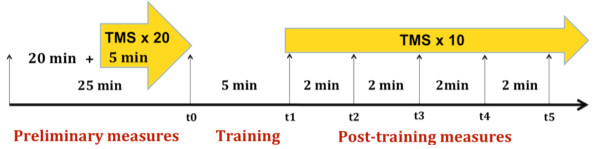
**Schematic summary of the experimental design**.

The preliminary phase of the experiment lasted about 25 minutes; with the electrodes and goniometer positioned as described above, the optimal site for stimulation and RMT were determined. The experimental design was structured in 3 phases: baseline, training, and post-training measurements (Figure 2). Subjects were comfortably seated with the right arm and hand relaxed in a vertical position, to avoid confounding gravitational contributions. This position was maintained throughout the experiment.

#### Baseline

Before training (at time-point t0), 20 TMS stimuli were delivered at 0.2 Hz to the optimal scalp site. Intensity of stimulation was calculated as the RMT intensity + 30% of MSO, to ensure large-size and easily measurable MEPs. Subjects usually perceived the twitch in the wrist, but not its direction, which was therefore indicated by the reading of the goniometer. Although the resultant movement induced by TMS would theoretically yield a vectorial combination of both sagittal and coronal deflection, all subjects exhibited a preferential plane of movement, thus explaining the choice to consider the dominant plane only.

#### Training

Once the baseline twitch direction in the dominant plane had been identified, subjects were instructed to perform voluntary phasic wrist movements in a direction opposite to it for 5 minutes at 0.2 Hz, as displayed on a monitor in front of the subject. The subjects performed one dynamic contraction through normal wrist movement range (extension or flexion) from the neutral position, followed by immediate relaxation, in which they were asked to let their wrist slowly and naturally drop, to allow passive return to neutral position. They were allowed 10 practice contractions to become familiar with the experimental setup. After each movement, we were able to monitor that the wrist returned to the start position by natural relaxation through visual feedback of the goniometrical traces. Accuracy and consistency of the direction of training exercises were monitored in real-time by the investigators throughout the experiment.

#### Post-training

At the end of the training period (at time-points t1 to t5), TMS was reapplied to the optimal site of motor cortex using the same parameters of stimulation, and subjects were tracked for 10 minutes, receiving 5 sets of 10 stimuli (at ~0.2 Hz), with a 2 minute delay between each set. Within each set, TMS pulses were separated by 5 seconds (50 seconds total at each time-point).

### Data Analysis

The outcome measures for this experiment were: 1) predominant direction of the TMS-induced movement twitch, indicated by the optical goniometer placed on the wrist; and 2) MEP amplitude for both FCR and ECR muscles, obtained through surface EMG recording and characterized during off-line analysis. For the goniometrical measurements of direction, we characterized changes in direction with a binary response by comparing consecutive pairs of time-points (t1 vs. t0, t2 vs. t1, etc.). For instance, '1' indicated a change in direction and sign, while a '0' was indicative of no change in direction and sign. We performed such comparison between all pairs of consecutive time-points and then analyzed whether there was a difference in the proportion of response across time-points. The data was analyzed using Fisher's exact test.

For the MEP amplitude, we conducted a mixed ANOVA model, with MEP amplitude as the dependent variable, and time-points and subject ID as independent variables. When appropriate we conducted post-hoc analysis with correction for multiple comparisons. Analyses were done with Stata^® ^statistical software (version 8.0, College Station, Texas).

## Results

### Muscle-Twitch Direction Change

**Figure 3:**
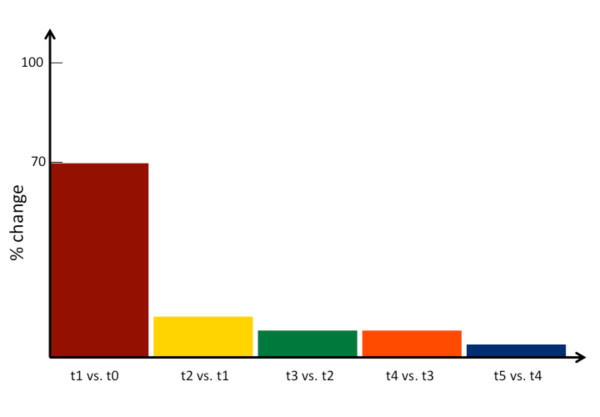
**Mean group data for change in twitch direction of the wrist, showing a significant effect *post *intervention at time-point 1, with ~70% of subjects having a reversed direction from the original twitch**. This effect was not sustained at time-point 2-5, and showed a trend to return to baseline across subjects by 10 minutes *post*.

Of the 20 subjects, 13 showed an initial and consistent TMS-twitch into flexion and thus trained into extension, while 7 subjects initially twitched into extension and trained into flexion. For the goniometer measurements treated as categorical data, the analysis performed across all time-points showed the change in direction to be maximal at the first time-point post training t1 compared to pre-training t0 (t1 vs. t0 = 70%, p < 0.01, percentage indicates percentage of subjects who changed direction), while the difference for each successive comparison was not significant: t2 vs. t1 = 15%, t3 vs. t2 = 10%, t4 vs. t3 = 10%, t5 vs. t4 = 5%; p > 0.05 (Figure 3). The difference between t1 vs. t0 remained significant until the last assessment at 10 minutes post intervention (p < 0.05 for the comparisons t2 vs. t0, t3 vs. t0, t4 vs. t0 and p = 0.06 for the comparison t5 vs. t0).

### Antagonist Muscle

**Figure 4:**
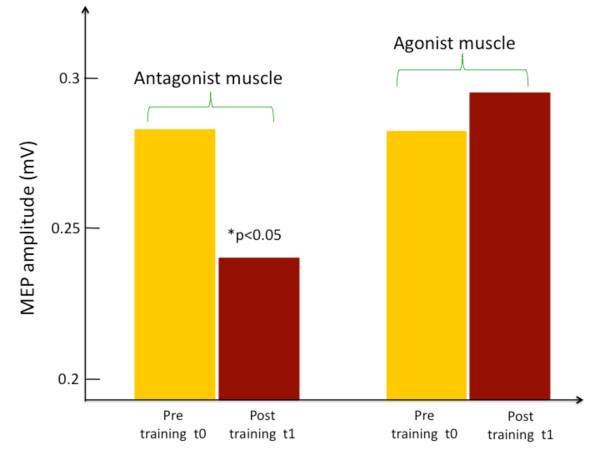
**Group MEP amplitude data (n = 20) recorded at rest before and immediately after training (t1)**. MEP amplitude in the antagonist muscle (to the trained muscle) was significantly reduced *post *training relative to *pre*, while the agonist (trained) muscle MEP amplitude was non-significantly elevated following the same training period.

**Figure 5:**
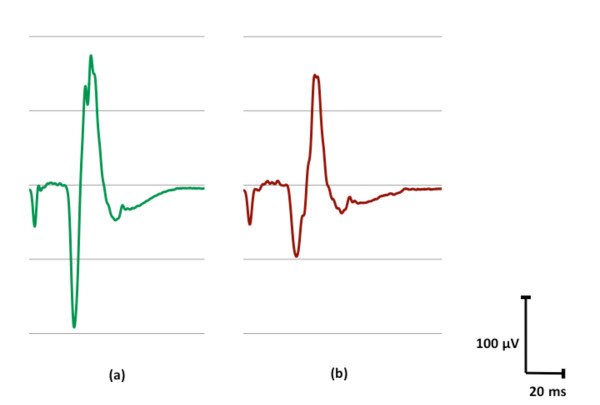
**Averaged MEP waveforms of one subject collected from the antagonist muscle at rest; (a) *pre *training and (b) immediately *post *training (t1), showing decreased amplitude following 5 minutes of training, associated with wrist movement, in direction opposite to that of original TMS-induced twitch**.

For the analysis of MEPs in the antagonist muscle, we observed a significant effect of time (F(5,95); p = 0.038)), suggesting that the training significantly affected MEP size in the antagonist muscle over time. Post-hoc analysis showed a significant difference in amplitude between the first time-point post training t1 and t0 (Figure 4): MEP amplitudes significantly decreased from 0.28 ± 0.05 mV at t0, to 0.24 ± 0.04 mV at t1 (p < 0.05). An example of such reduction taken from a single typical subject is presented in Figure 5, which shows averaged MEP waveforms collected from the antagonist muscle at rest (a) and following training (b). All the other comparisons were not significant (p > 0.05).

### Agonist Muscle

For the analysis of MEP amplitudes in the agonist muscle, the mixed ANOVA showed no significant differences in MEP for the main effect of time. Indeed, already at time-point t1 MEP amplitude was non-significantly elevated in the trained muscle, compared to t0 (t0 = 0.28 ± 0.07 mV, t1 = 0.29 ± 0.08 mV; F(5,95), p = 0.89), Figure 4.), suggesting that the training had no effect on the activity of the agonist muscle.

## Discussion

The present study demonstrated that five minutes of periodic, repetitive wrist movements were sufficient to invert the movement direction of the wrist generated by a TMS-induced muscle twitch. These direction changes were evident immediately post-training and progressively returned to baseline over the 10 minutes post-intervention. The change in twitch direction was associated with reduced cortico-motor excitability of the muscle opposing the trained direction, and did not depend on increased excitability in the agonist or trained muscle. Thus, these data suggest that early effects of repetitive non-skilled practice, considered to involve short-term plasticity in primary motor cortex, may involve release of constraining antagonist muscle activation.

It is well known that repetitive motor performance and skill learning result in functional organization of the human corticomotor system. The primary motor cortex can reorganize during recovery from lesion and motor skill acquisition [14-19], through unmasking of latent synapses [17] and modification of synaptic strength, including long-term potentiation mechanisms [20]. Numerous TMS studies have demonstrated that motor practice, skill acquisition and learning are associated with an increase in target muscle cortical excitability and a modulation of intracortical inhibition, but the relationship of cortical excitability changes with specific behavioural outcomes remains unclear [21].

Classen and colleagues showed that simple voluntary movements of the thumb repeated for a short time lead to a transient change in direction of a TMS-evoked twitch, towards the direction of training [13]. This suggests that the unskilled repetition of movements is sufficient to induce a reorganization of the neural network in M1 that encodes, at least in the short term, specific kinematic aspects of the practiced action. This experimental paradigm was also used to investigate use-dependent plasticity in subjects pre-medicated with drugs that influence synaptic plasticity [22]. Training was shown to evoke a relatively specific increase in cortical excitability for muscles mediating movements in the training direction, and a decrease in cortical excitability for muscles mediating movements in the baseline direction. This effect lasted for at least 30 minutes. Similarly, when learning-related changes in M1 excitability were studied with subjects who practiced either a ballistic or a ramp pinch task, an increase in force and acceleration, associated with an increase in MEP amplitude, was observed in the muscle involved in the training, but not in a muscle unrelated to the task. While MEPs returned to their baseline amplitude after subjects had acquired the new skill, no practice-induced changes in MEP amplitude were observed after subjects had over-learned the task, or after practicing a different task [23].

The principal difference between our study and the original work describing changes occurring with ballistic movements in the thumb, is that movements in the present study were 'steady and controlled' rather than 'brisk', as well as less frequent (0.2 Hz versus 1 Hz). Brisk movements require more synchronous activation of motor units to overcome limb inertia and accelerate the limb. It is interesting to note that both brisk and slow-to-moderate speed movements appear to yield a similar effect. Another difference in our study is the use of a biphasic TMS pulse, which is thought to recruit a larger population of cortical interneurons and consequently produce a greater MEP response than monophasic stimulation. Both forms of stimulation lead to multiple I-waves however [24], and our findings support the original paper by Classen and colleagues using a monophasic pulse, to suggest that this phenomenon is robust with both waveforms.

The precise mechanism of reduced antagonist muscle excitability cannot be elucidated from the present experiment. One possible explanation for the decreased antagonist excitability could be that M1 map expansion of the trained muscle could potentially result in cortical competition with surrounding muscle representations [25], which might include the antagonist muscle, however this is more likely to occur with skill training than simple repetition [3,26,27]. Similarly, the role of local intracortical excitability changes is unclear in relation to this type of practice. It is plausible that altered intracortical inhibition influences the evoked response amplitude, since this may be implicated with motor practice [28-30] but would need to be tested with the present protocol. The repetitive activation in our study involved agonist muscle activation only, since gravity returned the limb to the starting position. The antagonist participated passively, undergoing repeated passive lengthening and shortening. Our previous work shows that passive muscle lengthening alone can profoundly reduce cortico-motor excitability as the muscle undergoes lengthening, yet these effects are typically not sustained longer than the movement itself, and thus are unlikely to contribute to these results [31,32]. Furthermore, we might not consider the antagonist muscle to be purely passively involved during this protocol (such as when an external device is responsible for the cyclic back and forth movement). The precise mechanism of reciprocal inhibition in spinal circuits controlling wrist muscles is complex and unclear pertaining to our findings [33], however we expect that coupled with the repetitive descending voluntary drive to the agonist muscle, is local or descending inhibition to antagonist muscles through spinal interneurons [1,34,35]. Repeated net inhibitory activity of the antagonist corticospinal pathway may lead to a short-term sustained effect such as that observed in the present study.

Another important consideration is the possibility that the short-term plasticity we observed shares a spinal, as well as cortical component. Previous findings of rapid plasticity using a similar training paradigm were attributed to changes at the level of the cortex [13,23], based on electrical stimulation experiments [36], however potential spinal excitability changes cannot be ruled out in the present study. Further studies are necessary to probe specific cortical *and *spinal inhibitory mechanisms underlying this phenomenon, including quantification of spinal excitability such as H-reflex or F-wave measurement.

Whether reduced antagonist muscle excitability would be present during typical motor rehabilitation or skill training protocols involving alternating flexion-extension movements, is unclear. Our findings highlight the importance of considering the *nature *of the repetitive practice, which may become particularly pertinent for contemporary rehabilitation protocols combining non-invasive brain stimulation with repetitive motor training. In fact such protocols aim to augment the sustained changes in synaptic efficacy brought about through training, by altering motor cortex excitability during or before training. Repetitive motor skill practice (but not passive training), transiently increases motor cortex excitability and reduces cortical inhibition [28,37]. These transient changes in excitability can lead to sustained, cumulative changes, and are associated with motor learning [19]. Interventions such as transcranial direct current stimulation (tDCS) that enhance motor cortex excitability and reduce cortical inhibition are therefore appealing for augmenting motor learning in behavioral therapies [38-40]. Here we present data supporting the idea that depending on the nature of the training and role of specific muscles, these may be affected differently, and perhaps differentially interact with tDCS. The implication for the present findings is that muscles are likely to be differentially affected with excitability changes according to the specifics of the training.

While there is evidence indicating that behaviorally driven functional plasticity is a characteristic feature of motor cortex, and that motor behaviour associated with skill learning is crucial in shaping the functional organization of M1 [27], further investigation on how simple motor use may contribute to the production of short-term plasticity in M1, as shown in the present study, is needed. In a much broader framework, it is plausible to be able to exploit these transient plastic changes in the neuro-rehabilitation context (for example in stroke and hypertonic disorders), where there is maladaptive plasticity resulting in inefficient muscle activation, and potential to promote restoration of movement control.

A limitation of the present study design was the lack of power to conduct a multi-factorial analysis that includes all the data (i.e., agonist and antagonist muscle data); therefore future studies with a larger sample size should be conducted to confirm the results of this study.

## Conclusions

A single session of repeated wrist movements is sufficient to transiently alter the response to a TMS-induced muscle twitch direction. Movement direction changed to match the direction of practice, opposite to the original twitch. This direction change was accompanied by a reduction in corticospinal output to the muscle antagonistic to the trained direction, with no significant increase in output to the trained muscle.

The present study has proposed reduced activation of the antagonist muscle as a possible explanation for the change in direction of the TMS-induced muscle twitch, and demonstrated that this phenomenon can be evident in forearm muscles controlling the wrist. It remains to be determined if other muscles, in the upper or lower extremities, can exhibit the same behavior, and whether the same patterns of muscle activation can be observed in joints that have a less defined agonist/antagonist relationship. Future studies should consider varying the different parameters of this experiment, to see whether the effects can be modulated. Particular attention to the number of repetitive movements, frequency and speed at which they should be performed, and the possibility of extending the training over time, is relevant in determining the optimal parameters to maximize the magnitude and duration of the observed effects. The effect of ballistic versus smooth and slow movements could be compared, and how the results might differ in patient populations such as stroke, where extensor muscle weakness and flexor spasticity might influence the response.

Our results suggest that initial patterns of motor activity may be encoded in the corticospinal system with movement repetition of the wrist, consistent with an early phase of learning, and involve release of activation to antagonist muscles. These findings may have implications for training paradigms in the neurorehabilitation field.

## Competing interests

The authors declare that they have no competing interests.

## Authors' contributions

DJE conceived the study and contributed to writing the manuscript, VG carried out the experiments, collected results and wrote the manuscript, FF selected and performed the statistical analysis, BTV participated in the design of the study and helped to draft the manuscript, GT, APL and HIK helped to draft the manuscript and contributed to the revision. All authors read and approved the final manuscript.

## Acknowledgement

This work was supported by NIH grant 1R21HD060999-01 for DJE
